# Quantitative Evaluation of Successful Combination Therapy With Dye Laser and Long‐Pulse Alexandrite Laser for Papulopustular Rosacea

**DOI:** 10.1111/1346-8138.17928

**Published:** 2025-08-23

**Authors:** Reiko Nakamura, Motoki Nakamura, Kayoko Okuda, Sachiyo Takagi, Akimichi Morita

**Affiliations:** ^1^ Department of Geriatric and Environmental Dermatology Nagoya City University Graduate School of Medical Sciences Nagoya Japan; ^2^ Anti‐Aging Leser Care Center Nagoya City University Mirai Kousei Hospital Nagoya Japan

**Keywords:** alexandrite laser, combination therapy, dye laser, papulopustular rosacea, rosacea

## Abstract

Rosacea is a chronic skin disease characterized by facial flushing, dilated blood vessels, rashes, and swelling and has a significant impact not only on the patient's appearance, but also on their quality of life. Dye laser has the effect of contracting dilated blood vessels by selectively being absorbed in oxidized hemoglobin, and as a result, it improves flushing and dilated blood vessels. Dye laser is a useful option for erythematotelangiectatic rosacea (ETR). However, its effectiveness for papulopustular rosacea (PPR) is limited, and it is not recommended in several national guidelines. We examined the treatment effects of 28 cases diagnosed with rosacea and treated with dye laser therapy at Nagoya City University Hospital from April 2019 to March 2023. The cohort comprised 6 males, 22 females, 13 cases of ETR, 12 cases of PPR, and 3 cases of phymatous rosacea (PhyR). The evaluation was based on clinical photographs, and the redness and blueness of the skin were deconvoluted and quantified using ImageJ/Fiji software. In the case of ETR, there was a significant improvement between before and after treatment (*p* = 0.039). In the case of PPR, there was no significant improvement in the five cases treated with dye laser alone (*p* = 0.083). Still, there was significant improvement in the seven cases treated with a combination of long‐pulse alexandrite (LPA) laser (*p* = 0.0024). A multiple logistic regression analysis was performed on the combined therapies other than LPA laser, including topical corticosteroids, tacrolimus, metronidazole, sulfur and camphor, azelaic acid, antibiotics, or vitamin B. No treatment was found to contribute to significant improvement. LPA laser is expected to reduce inflammation around hair follicles seen in PPR effectively, and its combination with dye laser is a promising treatment option for PPR. Furthermore, this quantitative method based on clinical photographs is useful for the objective evaluation of rosacea treatment.

## 1 Introduction

Rosacea is a common chronic inflammatory skin disease that primarily affects the central facial region, presenting with symptoms such as persistent facial redness, dilated blood vessels, swelling, and in certain cases, inflammatory lesions. While rosacea can occur in individuals of any age, it is particularly prevalent among middle‐aged and older women, and it is often classified into subtypes based on its clinical features. These subtypes include erythematotelangiectatic rosacea (ETR), papulopustular rosacea (PPR), phymatous rosacea (PhyR), and ocular rosacea, each characterized by distinct symptoms [1, 2, 3, 4, 5, 6]. The psychological and social impact of rosacea can be substantial, as facial redness and other visible symptoms often lead to self‐consciousness and a reduction in quality of life. Effective management of rosacea is therefore essential not only for addressing physical symptoms but also for improving overall patient well‐being [7, 8].

Laser therapy has emerged as a valuable treatment option for managing ETR, especially for reducing facial redness [9, 10]. Dye lasers, which selectively absorb oxidized hemoglobin in blood vessels, are particularly effective in treating ETR as they directly target dilated blood vessels, leading to visible reductions in facial flushing and redness. Despite these benefits for ETR, the use of dye laser therapy for PPR has shown limited efficacy, as many national treatment guidelines do not currently recommend laser therapy for PPR [6, 11, 12, 13]. This limitation is attributed to the inflammatory nature of PPR, which may not respond adequately to treatments aimed solely at reducing vascular dilation. For PPR, standard treatments often include topical and oral anti‐inflammatory agents such as metronidazole and doxycycline, which target the inflammatory lesions rather than the vascular components. A major challenge in expanding the use of laser therapy other than dye laser for rosacea including PPR lies in the lack of standardized, objective methods to evaluate treatment efficacy.

In this study, we aimed to quantitatively evaluate the treatment effects of dye laser therapy and in combination with long‐pulse alexandrite (LPA) laser in patients with rosacea. Over a four‐year period, we examined 28 cases of rosacea treated at our hospital, with a specific focus on patients with ETR and PPR. Using ImageJ software, we developed a method to separate and quantify changes in skin redness and blueness from clinical photographs, offering an objective approach to assess treatment outcomes. This approach allowed us to standardize the evaluation of laser therapy effectiveness across different rosacea subtypes. By providing a quantitative assessment, we hope to clarify the potential of combined laser therapy as a treatment option for PPR and introduce a practical, reproducible evaluation method that may contribute to improved management and study of rosacea.

## 2 Methods

### 2.1 Patient Cohort

Based on the IRB approval (Nagoya City University Clinical Trial Management Center, No. 60‐24‐0048), the cohort consisted of 28 patients who were diagnosed with rosacea and underwent dye laser therapy at Nagoya City University Hospital between April 2019 and March 2023. The breakdown was 6 males and 22 females, with an average age of 47.9 years; 13 cases of ETR, 12 cases of PPR, and 3 cases of PhyR (Table 1). In the Fitzpatrick skin phototype (FSPT), there were 13 cases of type II, 11 cases of type III, and 4 cases of type IV. In the Investigator's global assessment (IGA) score, there were 16 cases of score 2 and 12 cases of score 3.

**TABLE 1:** Patient cohort.

Characteristics	Value
Cases	28
Age (range)	47.9 (29–80)
Sex	
Male	6
Female	22
Type	
ETR	13
PPR	12
PhyR	3
FSPT	
II	13
III	11
IV	4
IGA	
1	0
2	16
3	12
4	0

Abbreviations: ETR, erythematotelangiectatic rosacea; FSPT, Fitzpatrick skin phototype; IGA, investigator's global assessment; PhyR, phymatous rosacea; PPR, papulopustular rosacea.

### 2.2 Laser Treatments

Dye laser irradiation was performed using the Vbeam 595 nm pulsed dye laser (Candela, Wayland, MA, USA) with a 7 mm spot, 20 msec, 8–11 J at intervals of about 4 to 6 weeks. The GentleLASE Pro 755 nm LPA laser (Candela) with a 15 mm spot, 3 msec, 16 J in the laser facial mode was used after 40 min of cooling following dye laser irradiation in some PPR cases. Unlike previous reports, our cohort found that laser treatment for PPR was significantly effective. This was because we used the LPA laser in some cases, as part of a combination of treatments that included the dye laser. No local anesthesia, including lidocaine cream, was administered prior to either laser treatment.

### 2.3 Other Treatments

For antibiotic treatment, 100 mg of either doxycycline or minocycline was administered daily. As vitamin B, 20 mg of riboflavin and 30 mg of pyridoxal were administered as the daily dose. Sulfur and camphor (SC) lotion, topical metronidazole (MNZ) gel (Rozex, Maruho, Osaka, Japan), and oxytetracycline hydrochloride and hydrocortisone as a topical corticosteroid (CS) were applied to the affected area twice a day. DRX Azelaic acid (AZA) cream (Rohto Pharmaceutical, Osaka, Japan) and topical tacrolimus (TCL) were applied once a day.

### 2.4 Image Analysis

**FIGURE 1:**
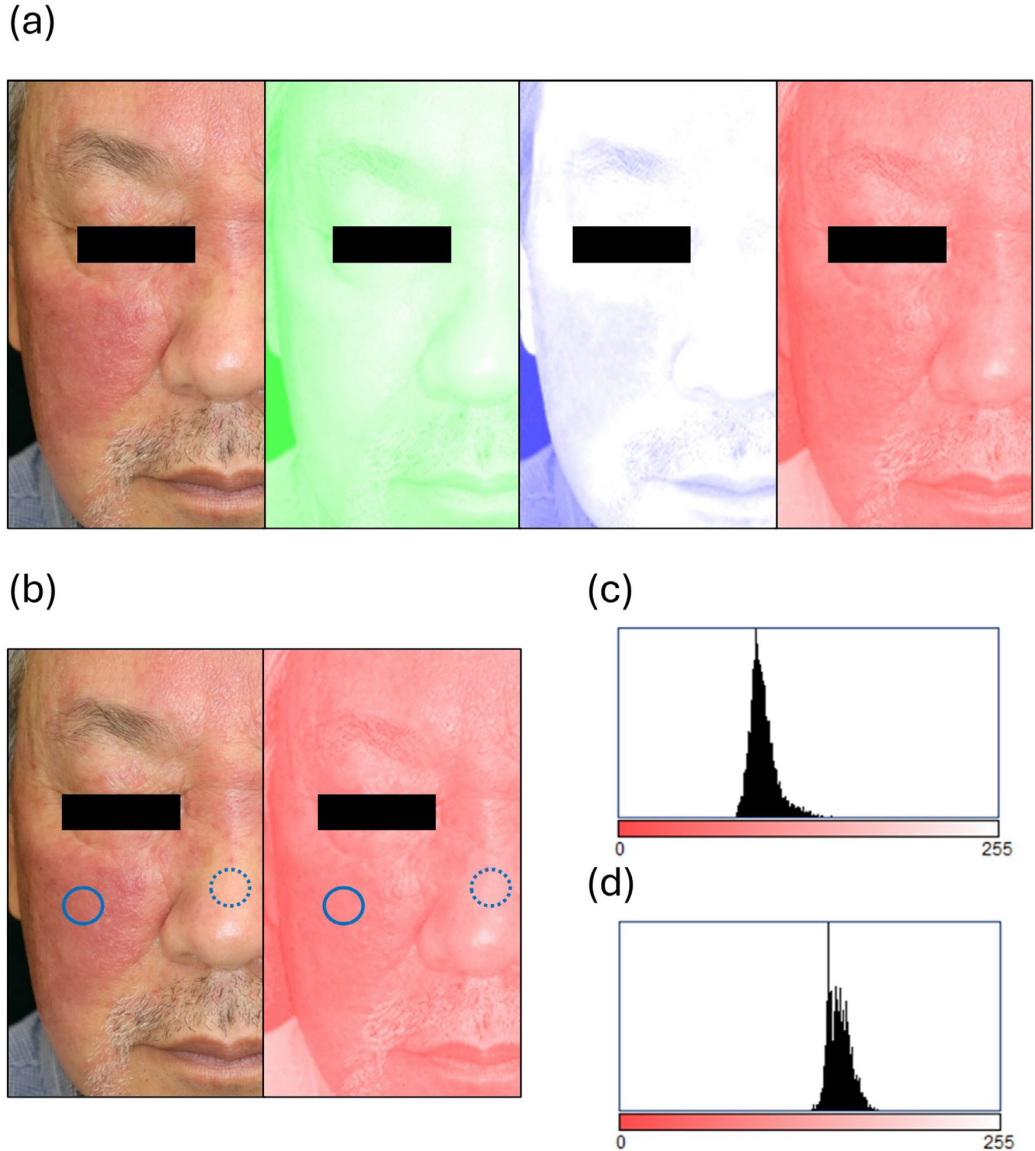
(a) Color deconvolution to split clinical photographs into the three colors of RGB (red, green, blue). (b) Solid circles indicate lesional areas, and broken circles indicate normal areas. (c) The histogram shows the luminance of the lesional area. The average luminance value was 96.996. (d) The histogram shows the luminance of the normal area. The average luminance value was 147.456.

Clinical photographs were analyzed using the open‐source software ImageJ/Fiji (NIH, Bethesda, MD, USA). Color deconvolution was performed to split clinical photographs into the three colors of RGB (red, green, blue, Figure 1a), and the average luminance values of red or blue were measured for three lesional and three normal areas (Figure 1b–d). Zero of the luminance value is the reddest, and 255 of the luminance value is white. The relative luminance values were calculated by dividing the luminance values of the lesional areas by the luminance values of the normal areas. If the relative luminance value is less than 1, it is redder or bluer than the normal area. The improvement in redness or blueness was evaluated based on the ratio of the relative luminance values before and after treatment. We defined an improvement as a relative luminance value of 1.1 or more.

### 2.5 Statistical Analysis

Statistical analyses were performed using Graph Pad Prism 9 (Graph Pad Software, San Diego, CA). Probability values of less than 0.05 were considered statistically significant.

## 3 Results

### 3.1 Quantitative Evaluation of Laser Treatment for Rosacea

**FIGURE 2:**
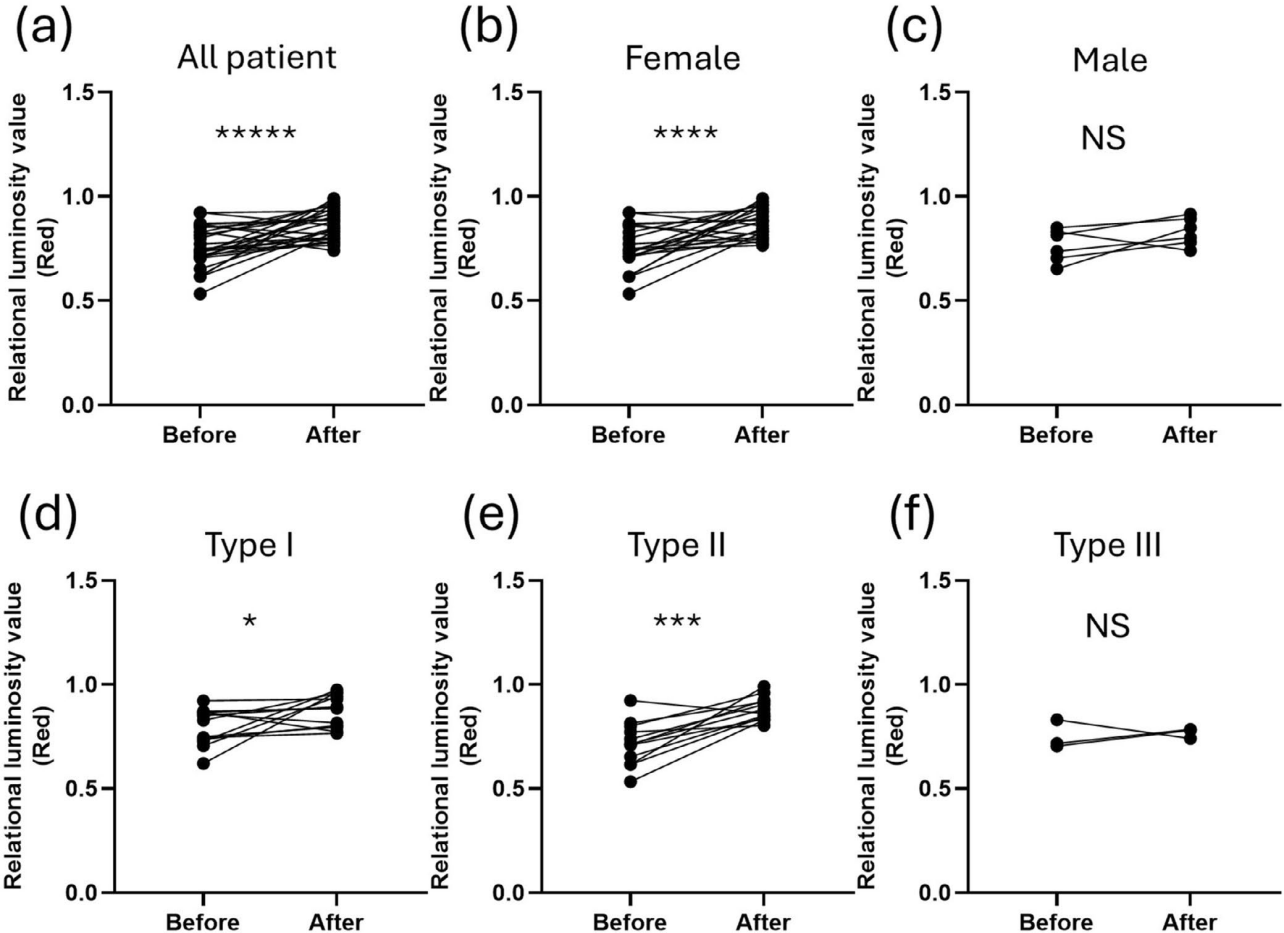
(a) The relative redness luminance values are compared before and after treatment in all cases (*n* = 28, *p* < 0.0001). (b) There is a significant difference in improvement among females (*n* = 22, *p* = 0.0004). (c) There is no significant difference among males (*n* = 6, *p* = 0.219). (d) Significant improvements are seen in ETR (*n* = 13, *p* = 0.027, average number of sessions = 4.2). (e) Significant improvements are seen in PPR (*n* = 12, *p* = 0.0015, average number of sessions = 4.3). (f) No improvement is seen in PhyR (*n* = 3, *p* = 1.00). All paired tests were analyzed by the Wilcoxon matched‐pairs signed rank test. *p ≤ 0.05, ***p ≤ 0.001, ****p ≤ 0.0001, *****p ≤ 0.00001, N.S., not significant.

The redness of the skin was quantified based on clinical photographs, and the results were compared before and after treatment. In all 28 cases, significant improvements were seen before and after treatment (*n* = 28, *p* < 0.0001, Wilcoxon signed rank test, Figure 2a). While there was a significant difference in improvement among females only (*n* = 22, *p* = 0.0004, Figure 2b), there was no significant difference among males only (*n* = 6, *p* = 0.219, Figure 2c). In the evaluation by type, significant improvements were seen in ETR (*n* = 13, *p* = 0.027, average number of sessions = 4.2, Figure 2d) and PPR (*n* = 12, *p* = 0.0015, average number of sessions = 4.3, Figure 2e), but no improvement was seen in PhyR (*n* = 3, *p* = 1.00, Figure 2f).

### 3.2 Additional Long‐Pulse Alexandrite Laser for Papulopustular Rosacea

**FIGURE 3:**
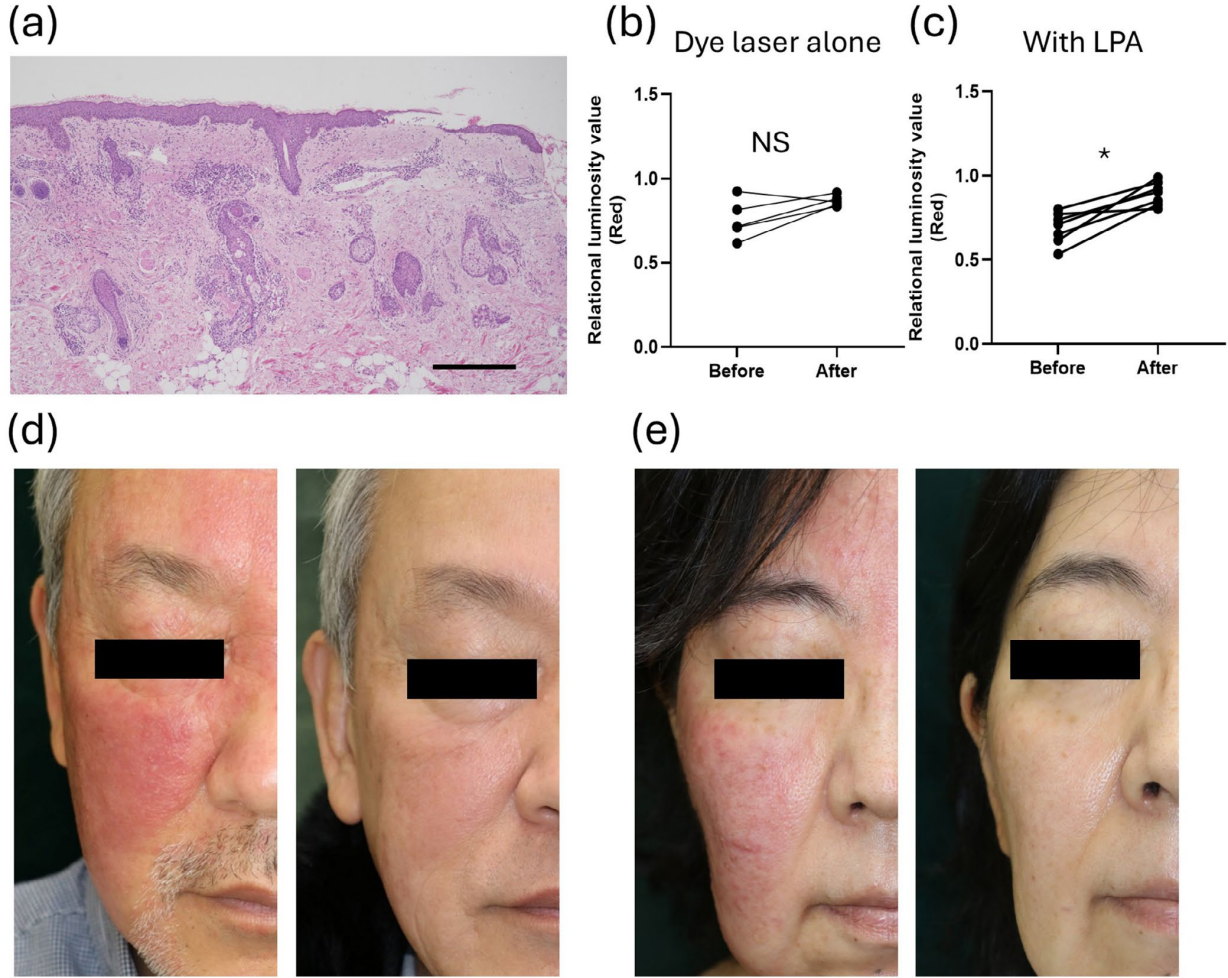
(a) Pathological image of PPR. scale bar, 500 μm. (b) PPR cases treated with dye laser alone do not show significant improvement (*n* = 5, *p* = 0.13). (c) PPR cases treated with dye laser and LPA laser in combination shows significant improvement (*n* = 7, *p* = 0.016). All paired tests were analyzed by the Wilcoxon matched‐pairs signed rank test. *p ≤ 0.05, N.S., not significant. (d, e) Clinical photographs of representative cases. The left panel is before treatment, and the right panel is after treatment.

In contrast to previous reports, our cohort showed a significant effect of laser treatment on PPR. This is because we used not only a dye laser but also an LPA laser in some cases. The pathological image of PPR is characterized not only by dilated capillaries in the upper middle layer of the dermis but also by inflammatory cell infiltration around the hair follicles (Figure 3a). The size of the dilated blood vessels in the upper layer of the dermis is around 100–200 μm, and improvement can be expected with a pulse width of 20 msec with dye laser treatments; however, it is not effective for inflammation around the hair follicles. Therefore, the LPA laser at 755 nm was added to target the hair follicles. PPR cases treated with the dye laser alone did not show significant improvement (*n* = 5, *p* = 0.13, Figure 3b), but cases treated with the dye laser and the LPA laser in combination did show significant improvement (*n* = 7, *p* = 0.016, Figure 3c). Clinical photographs of representative cases are shown. The case in Figure 3d is a 64‐year‐old male with FSPT III and an IGA score of 3 before treatment. He underwent three sessions of dye laser treatment and four sessions of LPA laser treatments. In addition to laser treatment, he was also taking oral doxycycline and vitamin B. The case in Figure 3e is a 57‐year‐old female with FSPT III and an IGA score of 3 before treatment. She underwent three sessions of dye laser and LPA laser treatment. As a combination therapy, she was taking oral doxycycline and vitamin B and using topical MNZ and SC lotion.

### 3.3 Quantitative Evaluation of Other Treatments for Rosacea

**FIGURE 4:**
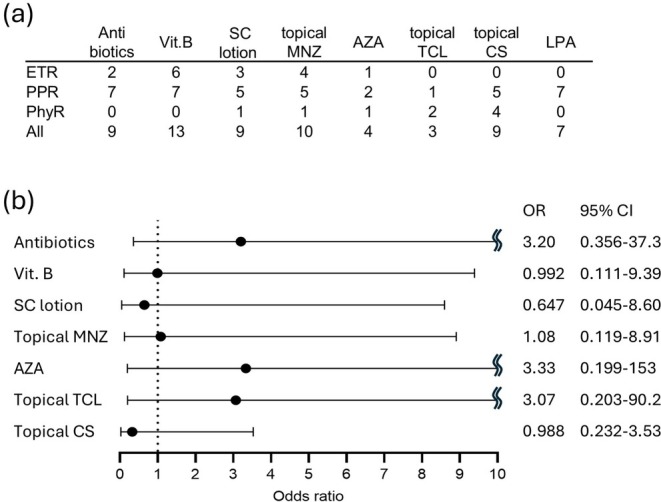
(a) A table shows how LPA laser and other therapies were used in combination with the dye laser for each type of rosacea. ETR, erythematotelangiectatic rosacea; PPR, papulopustular rosacea; PhyR, phymatous rosacea; SC, sulfur and camphor; MNZ, metronidazole; AZA, azelaic acid; TCL, tacrolimus; CS, corticosteroid; LPA, long‐pulse alexandrite laser; (b) A multiple logistic regression analysis was performed on the combined therapies other than LPA laser, with a pre‐post ratio of relative luminance values of 1.1 or more as an improvement. OR, odds ratio; CI, confidence interval.

Laser therapy was performed in combination with several oral and topical therapies. Figure 4a summarizes how LPA laser and other therapies were used in combination with the dye laser for each type of rosacea. A multiple logistic regression analysis was performed on the combined therapies other than LPA laser, with a pre‐post ratio of relative luminance values of 1.1 or more as an improvement. No combination treatment was found to contribute to improvement with a significant difference (Figure 4b).

### 3.4 Evaluation of Blueness

**FIGURE 5:**
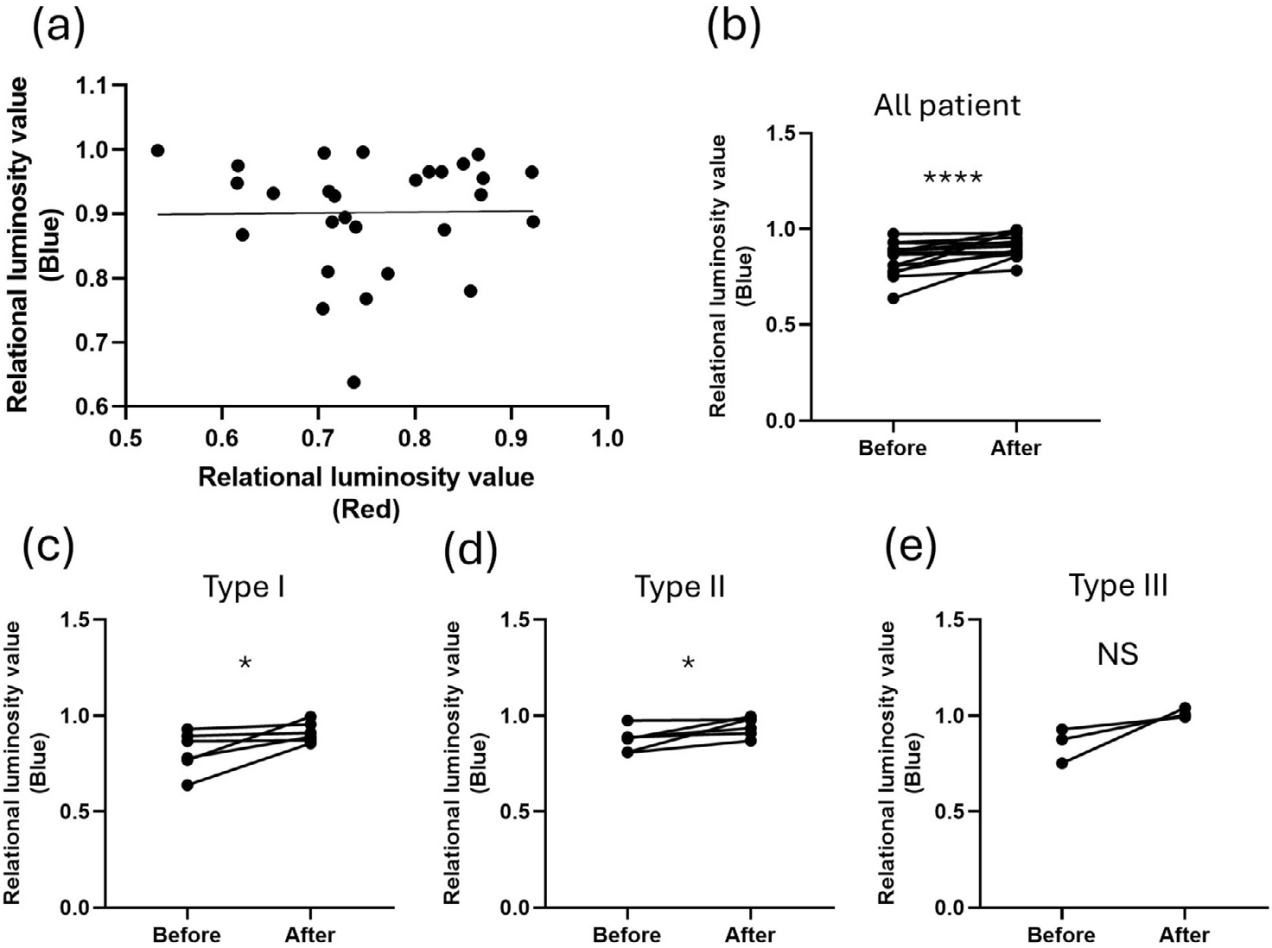
(a) There is no correlation between the luminance values of redness and blueness (*p* = 0.94, linear regression). (b) Significant improvements are observed in the comparison of the relative luminance value of blueness before and after treatment in all cases (*n* = 28, *p* = 0.0004). (c) Significant improvements are seen in ETR (*n* = 13, *p* = 0.031). (d) Significant improvements are seen in PPR (*n* = 12, *p* = 0.031). (e) No improvement is seen in PhyR (*n* = 3, *p* = 0.25).

As with redness, blueness was quantified and evaluated in the same way after color deconvolution. There was no correlation between the luminance values of redness and blueness (*p* = 0.94, linear regression, Figure 5a). Comparing all cases before and after treatment, there was also a significant improvement in the blueness (*n* = 28, *p* = 0.0004, Wilcoxon signed rank test, Figure 5b). In the evaluation by type, significant improvements were seen in ETR (*n* = 13, *p* = 0.031, Figure 5c) and PPR (*n* = 12, *p* = 0.031, Figure 5d), but no improvement was seen in PhyR (*n* = 3, *p* = 0.25, Figure 5e).

## 4 Discussion

In this study, we proposed a method for evaluating the efficacy of rosacea treatment by color deconvolution and quantifying the redness and blueness of the skin from clinical photographs, and showed that combination therapy using a dye laser and LPA laser is effective for PPR, which was previously thought to be ineffective for laser treatment. There have been attempts to objectively evaluate skin color for the past long time [14, 15]. In recent years, various devices that quantify skin conditions have been developed and are becoming more widespread [16]; however, they are expensive and it was not possible to analyze past cases. This method can be analyzed from ordinary clinical photographs, and because it uses only free software, it does not cost extra. Retrospective analysis is possible as long as clinical photographs are available. One of the reasons for the lack of evidence for rosacea treatment was the difficulty of quantitative and objective evaluation, but this evaluation method will be a great help. The majority of clinical studies rely on subjective visual assessments, which can introduce variability and make it difficult to compare results across different studies or patient populations [17]. This objective, quantitative evaluation method can provide significant advantages by enabling more consistent measurement of changes in skin condition, potentially leading to more accurate assessments of treatment efficacy. In this quantitative method, we adopted the RGB color space for skin color evaluation. This decision was made based on the ease of conversion and the ease of understanding the evaluation of red and blue colors when treating vascular and inflammatory skin lesions. On the other hand, the CIELAB color space is considered to be more accurate than the RGB color space in that it expresses skin color in a form closer to human color perception [18, 19]. The application of this evaluation method to the CIELAB color space is also considered for future development.

Among the various subtypes of rosacea, ETR is characterized by persistent redness and visible blood vessels (telangiectasia) due to vascular dilation, while PPR involves inflammatory papules and pustules similar to acne, primarily around hair follicles. These clinical differences imply that optimal treatment approaches for ETR and PPR may vary, as the underlying pathophysiology differs. For instance, ETR symptoms are primarily vascular in origin, whereas PPR includes a significant inflammatory component. Consequently, treatment for rosacea is typically tailored to the subtype and may include a combination of topical, oral, and procedural interventions. Recent technological advancements and research in laser therapy, including LPA lasers, have opened new possibilities for addressing the inflammatory components of PPR [20]. LPA laser may not only act on telangiectasias but also calm down inflammation by eliminating hair follicles, which are the targets of inflammation in PPR [21]. The effects of LPA laser on the skin microbiome in rosacea have also been reported [22]. Recent reports have suggested that LPA laser may offer enhanced results in reducing redness in rosacea treatment [23], although clinical evidence remains limited for PPR. Adverse effects of LPA laser treatment include pigmentation, depigmentation, blistering, scar formation, and other effects, as well as reported cases of paradoxical worsening of acne and rosacea‐like dermatitis [24]. Although no cases with adverse effects were observed in our cohort, further studies with a larger number of cases are needed to evaluate the adverse effects of LPA laser treatment on PPR. The potential of such a combined approach in this study offers a promising direction for more effective management of rosacea, especially for PPR cases where single‐laser therapy has proven insufficient.

In conclusion, our findings based on the objective quantitative evaluation support the potential of combining long‐pulse alexandrite (LPA) laser with dye laser for the effective treatment of papulopustular rosacea (PPR), a subtype that has traditionally shown limited response to laser therapy. In our cohort, patients treated with dye laser alone did not exhibit significant improvement, whereas those who received the combined therapy showed marked and statistically significant reduction in skin redness and inflammation. In contrast, additional therapies such as topical agents and oral antibiotics did not contribute significantly to clinical improvement when analyzed through quantitative evaluation. The observed efficacy of LPA laser may be attributed to its ability to target perifollicular inflammation, a hallmark of PPR pathogenesis. Therefore, the combined use of dye and LPA lasers represents a promising therapeutic option that warrants further prospective validation. This approach may redefine the laser treatment strategy for rosacea and provide clinicians with a new pathway to optimize patient outcomes in PPR cases.

## Conflicts of Interest

The authors declare no conflicts of interest.

## Data Availability

The data that support the findings of this study are available on request from the corresponding author. The data are not publicly available due to privacy or ethical restrictions.
